# Fracture of the Patella

**Published:** 1890-03-15

**Authors:** 


### FRACTURE OF THE PATELLA.

Mr. Fletcher Horne, surgeon to the Barnsley Beckett Hospital, contributes to the *Lancet*, an article on the treatment of fracture of the patella by Mayo Robson's method. The treatment of transverse fracture of the patella has been a *pons asinorum* to the surgeon, as evidenced by the variety of methods adopted; from the inclined plane, pulley and weight, Malgaigne's hooks, to laying open the joint and wiring the fragments together. But Dr. Thomas, of Liverpool, has said (*vide* Retrospect, October 12th, 1889), that of all the important fractures presented to the surgeon the easiest to restore and repair is fracture of the patella. Mr. Horne does not, however, agree with this observation, and says that of all fractures it is the most difficult to get bony union. In a large number of cases a fibrous union is all that can be expected, and to obtain the shortest ligamentous union, possibly an osseous one, is the object. Mr. Horne relates a case of transverse fracture treated as follows: The limb was first bathed with bichloride of mercury solution (1 in 1,000). Two steel pins seven inches long were procured. One was passed through the tendon of the gudriceps extensor, just above the superior edge of the upper fragment, the second needle being passed in similar manner through the ligamentum patellæ. By traction the fragments of the bone were easily brought into apposition. A figure of 8 ligature was passed over the pins to keep the fragments of bone in position, and the sharp points of the pins cut off. The cut ends were guarded by cotton wool, and the joint encased in a plaster of Paris band. The temperature never rose above normal, and three weeks afrerwards a window was cut in the bandage large enough to withdraw the pins. A month afterwards the limb was relieved of the bandage, the fractured parts being found in good position. The limb was placed on Arnold's knee splint with extension screw, held in position by plaster of Paris bandage, and the patient allowed to go on crutches. In three more months the splint was removed, union appearing bony and complete. Some weeks afterwards the patient walked well, showing neither stiffness nor deformity. But Mr. Thomas asserts that under his treatment patients are enabled to go about their business immediately after the fracture is put up. We observed in the "Retrospect" of October 12th, that should Mr. Thomas's treatment prove as effectual in other hands, he will deserve thanks for a great surgical advance. We should be glad to hear more from Mr. Thomas.

